# Instrumented Mechanical Total Knee Arthroplasty Routinely Decreases the Medial Posterior Condylar Offset

**DOI:** 10.7759/cureus.96480

**Published:** 2025-11-10

**Authors:** Anton Lambers, Matthew T Mann, Dermot Collopy, Gavin Clark

**Affiliations:** 1 Surgery, The University of Western Australia, Perth, AUS; 2 Orthopaedics, Northeast Health Wangaratta, Wangaratta, AUS; 3 Orthopaedics, Royal Perth Hospital, Perth, AUS; 4 Orthopaedic Surgery, Perth Hip and Knee Clinic, Perth, AUS; 5 Orthopaedic Surgery, St John of God Subiaco, Murdoch, and Midland Hospitals, Perth, AUS

**Keywords:** arthroplasty, functional alignment, mechanical alignment, posterior condylar offset, robotic-assisted total knee arthroplasty, total knee arthroplasty technique, total knee arthroplasty (tka)

## Abstract

To balance a tight medial flexion gap in functionally aligned total knee arthroplasty (FA-TKA), the femoral component is often externally rotated to the posterior condylar axis (PCA) such that more bone is removed from the medial posterior femoral condyle than is replaced with prosthesis. A reduction in the posterior condylar offset (PCO) has been thought to cause decreased flexion and poor outcomes. We performed a sawbone investigation to quantify posterior femoral condylar resection depth in mechanically aligned TKA using conventional resection guides, including the variations that occur with different degrees of external rotation (ER) to the PCA.

Seven left synthetic knee sawbone models of the same size were placed in a stable external vice grip and had posterior condylar resections performed for varying degrees of ER. The first trial had the guide set at a neutral (0°) angle to the PCA. The guide was then set to 1.5° increments of increasing ER from neutral (1.5°, 3°, 4.5°, and 6° of ER) and then to 3° and 6° of internal rotation (IR) from neutral. The thickness of the saw blade was added to a caliper measurement of resected bone to give the total resection thickness. The difference between this measurement and the implant thickness was then calculated to assess the resultant change to PCO both medially and laterally.

Neutral and externally rotated femoral cuts all yielded a reduction in the medial PCO. In a "standard" 3° externally rotated cut, there were 10 mm of posterior condyle resected medially and 2.5 mm of posterior condyle resected laterally (including bone and cartilage). A neutral cut (8.7 mm) very closely approximated posterior implant thickness (8.5 mm), resulting in the restoration of native PCO.

This study provides valuable insights into the changes to PCO during instrumented mechanical TKA that can assist surgeons in considering resection depths when moving to robotic knee arthroplasty systems, where resection depths are preoperatively templated and displayed intraoperatively for the surgeon. A standard workflow with 3° of femoral ER routinely decreases the medial PCO by up to 1.5 mm or, in the case of full-thickness cartilage wear, up to 5 mm. Further research should examine the relationship between the deliberate reduction of PCO and patient functional outcomes in the context of alternative alignment philosophies in TKA.

## Introduction

Restoration of femoral anatomy and preservation of joint kinematics are key objectives in total knee arthroplasty (TKA). Traditionally, with instrumented mechanical alignment philosophies, 3° of femoral external rotation (ER) relative to the posterior condylar axis (PCA) is factored in during femoral component positioning to approximate the transepicondylar axis (TEA). This was considered the average deviation between the PCA and TEA, although studies have shown that variation in this is common [1]. Posterior condylar offset (PCO) is the distance by which the posterior femoral condyle extends beyond the posterior cortex of the distal femur, reflecting how far the femoral condyles project posteriorly relative to the femoral shaft, which helps maintain femoral rollback and knee flexion [2].

With the advent of robotic-assisted systems, newer alignment philosophies such as functional alignment (FA) have gained popularity. FA aims to resurface bone and implant components to restore the plane and obliquity of the joint line and minimize soft tissue release [3-6]. This approach individualizes component positioning based on the native soft tissue envelope and joint laxity patterns, often requiring ER of the femoral component to balance a tight medial flexion gap [3,7]. One method of achieving this in FA is to externally rotate the femoral component while pivoting from the lateral posterior condyle. This maintains lateral PCO, but leads to the deliberate reduction of medial PCO with resection depths of up to 14 mm (range: 9-14 mm), including bone and cartilage [1].

While this technique may facilitate flexion gap balancing, concerns have emerged that the under-restoration of the medial PCO could potentially alter femoral rollback and affect range of movement (ROM) [8-10]. A decrease in PCO has long been theorized to limit high flexion as the femur impinges on the tibial insert sooner in deep bending [11]. Studies have suggested a close correlation between PCO magnitude and knee joint function after TKA, with a postoperative decrease in PCO of greater than 3 mm leading to reduced postoperative ROM and a 10° loss of maximum knee flexion range [12-15]. Maintaining the PCO and limiting changes to between 0 mm and 2 mm are superior to other changes for joint function after TKA, as well as improving patient satisfaction postoperatively [13]. Together, these studies raise legitimate concerns that excessive posterior medial femoral condyle resection, which often occurs with FA techniques, may contribute to poorer clinical outcomes compared to previous instrumented mechanical alignment.

Despite these concerns, objective data quantifying posterior medial condylar resection in instrumented mechanical alignment TKA across varying degrees of femoral ER remain limited. Wuertele et al. addressed this using a computed tomography (CT) scan-based model, assessing virtual posterior condylar resection on 100 postoperative instrumented mechanically aligned TKAs [1]. They found medial condylar resection ranged from 9 mm to 14 mm, often exceeding implant thickness and suggesting reduced PCO. Additionally, Kahlenberg et al., using a sawbone model, investigated the posterior condyle resection of five TKA implant systems set at 3° ER [16]. They discovered that each system varied resection amount, by up to as much as 3 mm, and all systems resulted in posteromedial condyle resection greater than the thickness of the implant, with resections of 9.4-12.4 mm, representing a decrease in the medial PCO of 0.6-2.9 mm. This study didn't look at any rotation to the PCA outside of 3° ER and was also confounded by using different sized implants from each system's size range, which may have resulted in variability. Furthermore, any presence of cartilage wear can asymmetrically influence the rotational guide alignment, with cartilage thickness in the posterior condyles being as high as 3.5 mm [17].

The aim of the present study was to assess the effect of different femoral rotation settings on the medial and lateral posterior femoral resections. Using a sawbone simulation of instrumented mechanical TKA, the primary objective was to quantify the resection thickness of the posterior medial femoral condyle at predefined degrees of ER. The secondary objective was to compare medial and lateral posterior condylar resections relative to the PCA. This biomechanical model provides a reproducible baseline for understanding how femoral rotation in mechanically aligned TKA compares to resections typically seen in robotic-assisted FA.

## Technical report

Study design and materials

This experimental study utilized seven synthetic femoral bone models to evaluate posterior medial condylar resection thickness at varying degrees of femoral component rotation. All models were identical in specification and clamped securely using a femoral head vice and a tibial stabilizing holder to ensure consistent positioning.

Instrumented mechanical alignment TKA was performed on each model using the Stryker Triathlon system (Stryker, Kalamazoo, Michigan, United States), employing an intramedullary (IM) femoral rod and standard cutting jigs. Distal femoral cuts were set to 5° of valgus and aimed for an 8 mm resection from the most prominent distal point. Femoral rotation was adjusted using a posterior condylar referencing guide in 1.5° increments, ranging from 6° internal rotation (IR) to 6° ER relative to the PCA.

Measurement tools

All dimensional measurements were conducted using CraftRight 150 mm metric vernier calipers (0.02 mm resolution; Bunnings Warehouse, Australia). Measurements were taken after bone resection, and the bone resected, in addition to the kerf thickness, was considered the total excision.

Procedure

**Figure 1:**
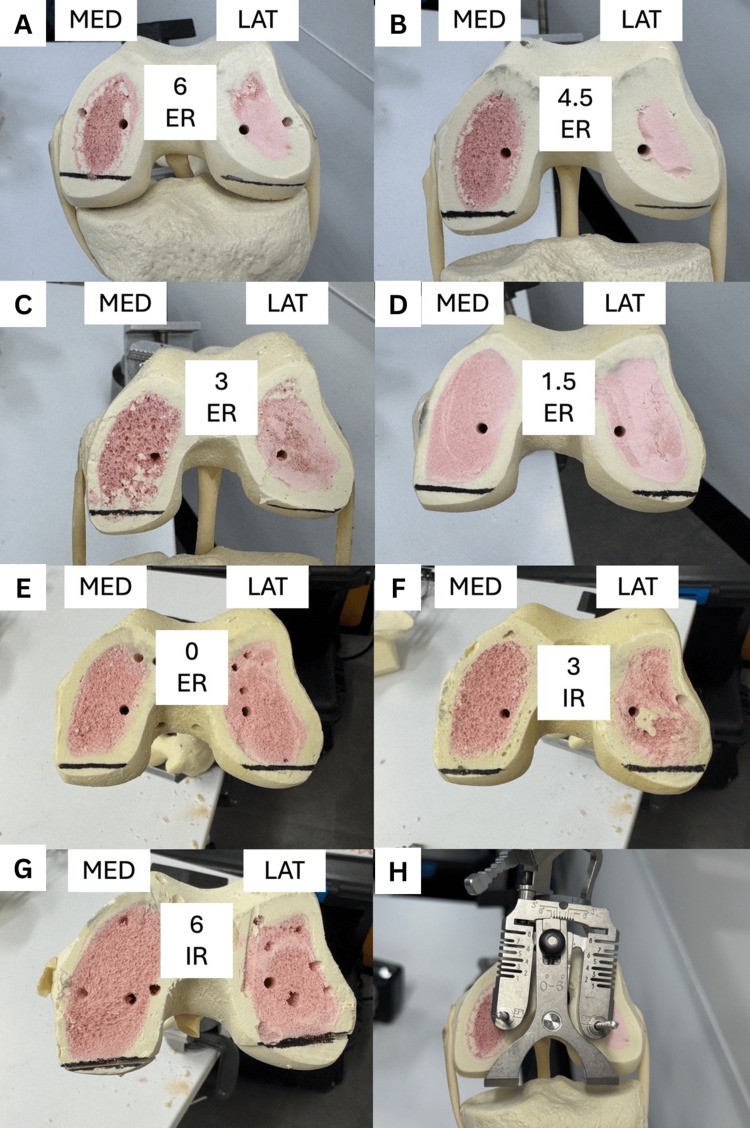
Photograph of the planned resections for each femoral rotation setting and the posterior-referencing guide for setting femoral rotation (A) 6° ER. (B) 4.5° ER. (C) 3° ER. (D) 1.5° ER. (E) 0° ER. (F) 3° IR. (G) 6° IR. (H) Posterior-referencing guide ER: external rotation; IR: internal rotation; MED: medial; LAT: lateral

**Figure 2:**
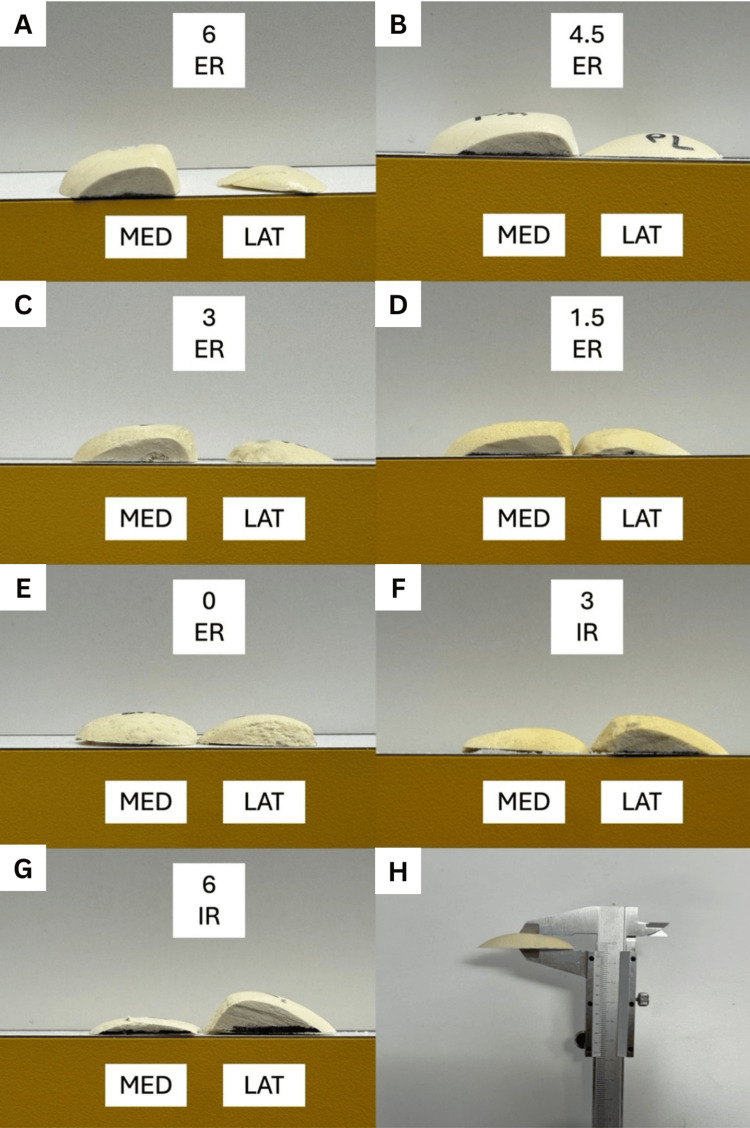
Photograph of the resections for each femoral rotation setting and the caliper technique for resection thickness (A) 6° ER. (B) 4.5° ER. (C) 3° ER. (D) 1.5° ER. (E) 0° ER. (F) 3° IR. (G) 6° IR. (H) Caliper technique for measuring resection thickness ER: external rotation; IR: internal rotation; MED: medial; LAT: lateral

For each specimen, the following steps were performed. Each femur was assigned a case number and clamped in the vice at the femoral head. A central IM entry point was drilled, and the distal femoral cutting block (5° valgus) was secured and cut at a 0 mm cut. The femoral rotation guide was then applied and set to a predetermined degree of rotation (from 6° IR to 6° ER) relative to the PCA. The posterior femoral cutting block (four-in-one guide) was placed accordingly. Planned posterior resections were marked with a surgical marker using the edge of the saw blade through the cutting block (Figure 1). Posterior femoral resections were performed. Resected bone thicknesses from the medial and lateral posterior condyles were measured directly. Side-on photographs of each posterior resection were taken with consistent orientation (left=medial; right=lateral) (Figure 2). All specimens were photographed together for visual comparison.

Results

**Figure 3:**
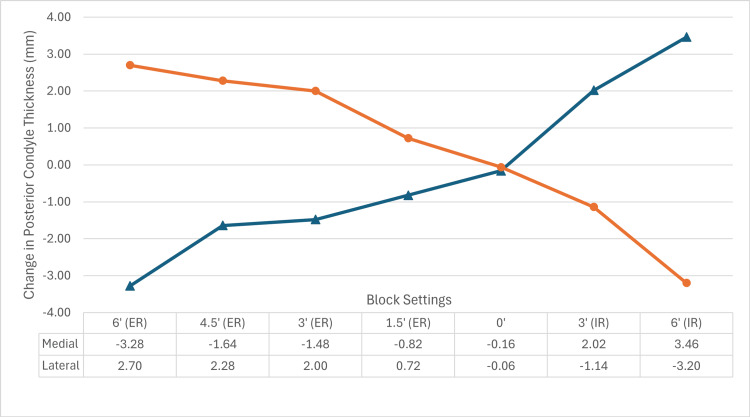
Trend in change to medial and lateral posterior condyle with varying degrees of rotation Block settings from 6° ER to 6° IR with change in posterior condyle thickness (mm) shown at each block setting. Medial posterior condyle change (triangle (∆)) and lateral posterior condyle change (circle (○)) represented individually in mm.

Three degrees of ER relative to the PCA resulted in a medial posterior femoral resection of 10 mm, resecting 1.5 mm more bone than is replaced with the implant system. As expected, the medial and lateral resection thickness changed in an inverse relationship, with medial resections increasing with ER (Figure 1). The maximum resections were 11.8 mm (medial, at 6° ER) and 11.7 mm (lateral, at 6° IR), corresponding to a post-implant offset change of -3.3 mm and -3.2 mm, respectively. The kerf of the blade measured 1.3 mm. A detailed summary of resections and post-implant PCO changes (based off an implant thickness of 8.5 mm) is presented in Table 1, and the trend PCO change is displayed in Figure 3.

**Table 1:** Posterior femoral condylar resection thickness (resected bone + kerf) and resulting post-implant offset changes across varying degrees of femoral component rotation relative to the posterior condylar axis Values are presented separately for the medial and lateral posterior femoral condyles. "Resection + kerf" represents the measured thickness including saw blade kerf (1.3 mm). "Post-implant change" indicates the difference between the resected bone and implant thickness (8.5 mm), with negative values reflecting under-restoration (i.e., decreased posterior condylar offset). ER: external rotation; IR: internal rotation

Block setting (degrees)	Medial posterior			Lateral posterior		
	Resected bone	Resection + kerf	Post-implant change	Resected bone	Resection + kerf	Post-implant change
6 (ER)	10.5	11.8	-3.3	4.5	5.8	2.7
4.5 (ER)	8.8	10.1	-1.6	4.9	6.2	2.3
3 (ER)	8.7	10	-1.5	5.2	6.5	2.0
1.5 (ER)	8	9.3	-0.8	6.5	7.8	0.7
0	7.4	8.7	-0.2	7.3	8.6	-0.1
3 (IR)	5.2	6.5	2	8.3	9.6	-1.1
6 (IR)	3.7	5	3.5	10.4	11.7	-3.2

## Discussion

Our results demonstrate that using a standard posterior-referencing technique with 3° of femoral ER leads to a relative over-resection of the medial posterior femoral condyle, resulting in a reduction in medial PCO. This finding aligns with Wuertele et al., who reported that standard guides (set at 3-7° of ER) produced medial posterior condylar cuts of about 9-14 mm, versus only 4-10.5 mm laterally [1]. Similarly, according to Kahlenberg et al., using sawbone models and five different implant systems set at 3° ER, the medial posterior femoral condyle resection averaged 9-12 mm [16]. This resulted in resection always exceeding implant thickness, reducing the PCO (range: 0.6-2.9 mm). For the first time, different femoral rotation settings with posterior referencing are quantified to give an indication of expected resection depths in vivo.

While FA-TKA often deliberately under-resects the medial posterior femoral condyle, the surgeon receives visual numeric feedback on this, which can be concerning. As demonstrated here, decreasing the medial PCO has been a longstanding practice in traditional TKA techniques and may have gone unnoticed for those who do not perform caliper checks of bone resections intraoperatively. The maintenance or increase in lateral PCO also needs to be considered when determining if decreased medial PCO will adversely affect the knee kinematics and outcomes. Further research is required to ascertain whether deliberate medial PCO reduction, as part of an FA-TKA technique, still encounters the reported poor outcomes, such as decreased ROM. When considering PCO, it is important to ensure a constant reference of depth is used. Joint position should be referenced to native cartilage. That was the reference used in this paper. Some available robotic systems utilize a subchondral bone reference based on CT imaging. This will result in a smaller resection depth by 2-3.5 mm.

The resection depths described here are a minimum, in particular on the medial side, where the posterior-referencing rotation guide may be placed on bone due to cartilage wear in that location. Cartilage in this area can be up to 3.5 mm thick before wear. Hence, the true medial posterior femur resection approximates 13 mm if a complete loss of articular cartilage was present at the time of surgery. Other factors that may increase the over-resection of posterior condyles include the use of minimally invasive cutting blocks that are narrower and potentially don't allow their feet to reach the most posterior part of the condyle or patients with large anatomy for similar reasons.

The strength of the study is the novel reporting of different rotational femoral cuts relative to the PCA, which can help bridge the gap in information for surgeons moving from instrumented to technology-assisted TKA. Knowing the resections that correspond to different rotations may aid with intraoperative decision-making and surgeon comfort, particularly during balancing. A limitation of our study is that, due to the in vitro nature of the study, the samples had little anatomical variation present, as well as a limited sample size, with only one sawbone femur model used per rotation setting. There is also a level of inherent inaccuracy in the reproducibility of resections, demonstrated by the minimal change (0.1 mm) in posterior medial resection between 3° and 4.5° of ER. Further studies on this topic could perform a similar series on instrumented TKAs on actual patients by recording caliper measurements of intraoperative cuts. Patient factors such as patient height and implant sizes could then be collected to examine their influence on resection thicknesses. Posterior condyle offset changes for the individual medial and lateral condyles could then be measured and assessed against patient outcomes postoperatively to investigate if decreased medial PCO, while maintaining the lateral PCO, impacts outcomes, such as ROM and patient satisfaction.

## Conclusions

This study provides valuable insights into the changes to PCO during instrumented mechanical TKA that can assist surgeons in considering resection depths when moving to robotic knee arthroplasty systems, where resection depths are displayed for the surgeon. A standard workflow with 3° of femoral ER has likely been routinely decreasing the medial PCO by up to 1.5 mm or, in the case of full-thickness cartilage wear, up to 5 mm. More research is encouraged to determine what magnitude of PCO reduction impacts patient outcomes.
